# Developing a measure to assess motivation and self-efficacy in anorexia nervosa using the Theory of Planned Behaviour

**DOI:** 10.1186/2050-2974-2-S1-O46

**Published:** 2014-11-24

**Authors:** Lisa Dawson, Barbara Mullan, Paul Rhodes, Stephen Touyz

**Affiliations:** 1 The University of Sydney, Sydney, Australia; 2 Curtin University, Perth, Australia

## 

Motivation in anorexia nervosa (AN) has been almost exclusively measured using the Transtheoretical Model of Change, despite criticisms of this model. In other areas of psychology, alternative models have been proposed to explain motivation. In Health Psychology, the Theory of Planned Behaviour (TPB) is a widely used and supported model. The aim of the current study was to determine the appropriateness of the TPB for understanding intentions to recover from AN and to develop a purpose-designed TPB measure to assess this. Thirteen adults who had recovered from AN were interviewed to elicit the salient beliefs underlying the three components of the TPB (attitudes, subjective norms, perceived behavioural control). Data from interviews revealed that beliefs and attitudes about recovery were compatible with the TPB and a 27-item self-report measure was developed. The TPB appears to be a good fit, from a theoretical perspective, for determining intentions to change in AN. The findings add to a growing body of research examining motivation in AN and provide further insight into how this can be measured.

This abstract was presented in the **Assessment** stream of the 2014 ANZAED Conference.

